# Light People: Professor Fanglin Bao and his cover story

**DOI:** 10.1038/s41377-024-01434-0

**Published:** 2024-05-22

**Authors:** Ji Wang

**Affiliations:** School of Science, Westlake University, Hangzhou, Zhejiang PR China

**Keywords:** Optical sensors, Optical physics

## Abstract

Qu Yuan, a renowned ancient Chinese poet, once pondered in his work *Heavenly Inquiry*: “If the sun’s light is absent, from where does the radiance of the Hero Flower emanate?” The ability to see clearly in the darkest of nights has long been a sought-after magical power by humans. It holds not only immense technological significance for computer vision and remote sensing but also profound implications for transcending the dichotomy between day and night in our daily lives. Professor Fanglin Bao from Westlake University has made significant breakthroughs in this field, bringing us closer to a world where we can transform night into day. His groundbreaking research on Heat-Assisted Detection and Ranging (HADAR) and night vision was featured as the cover story in *Nature* on July 26, 2023.

For this issue of “Light People”, Professor Fanglin Bao will share his research journey and the captivating story behind HADAR.

**Short Bio:** Fanglin Bao was born in Chun’an County, Zhejiang Province. He graduated from Zhejiang University in 2011 with a Bachelor’s degree in Physics and in 2016 with a PhD in Optics. He visited the Chinese University of Hong Kong in 2012, as an exchange scholar. He moved to Purdue University in 2019, as a postdoctoral scholar. He then became a research scientist at Purdue in 2021. He joined Westlake University in Jan. 2024 as an assistant professor. He proposed and demonstrated Heat-Assisted Detection and Ranging (HADAR), where he explained and overcame the ghosting effect in traditional thermal imaging. HADAR was highlighted as a *Nature* cover article^1^ and was reported by “*Nature* News & Views”, *Science* “News”, *Nature Photonics* “News & Views”.

**Figure:**
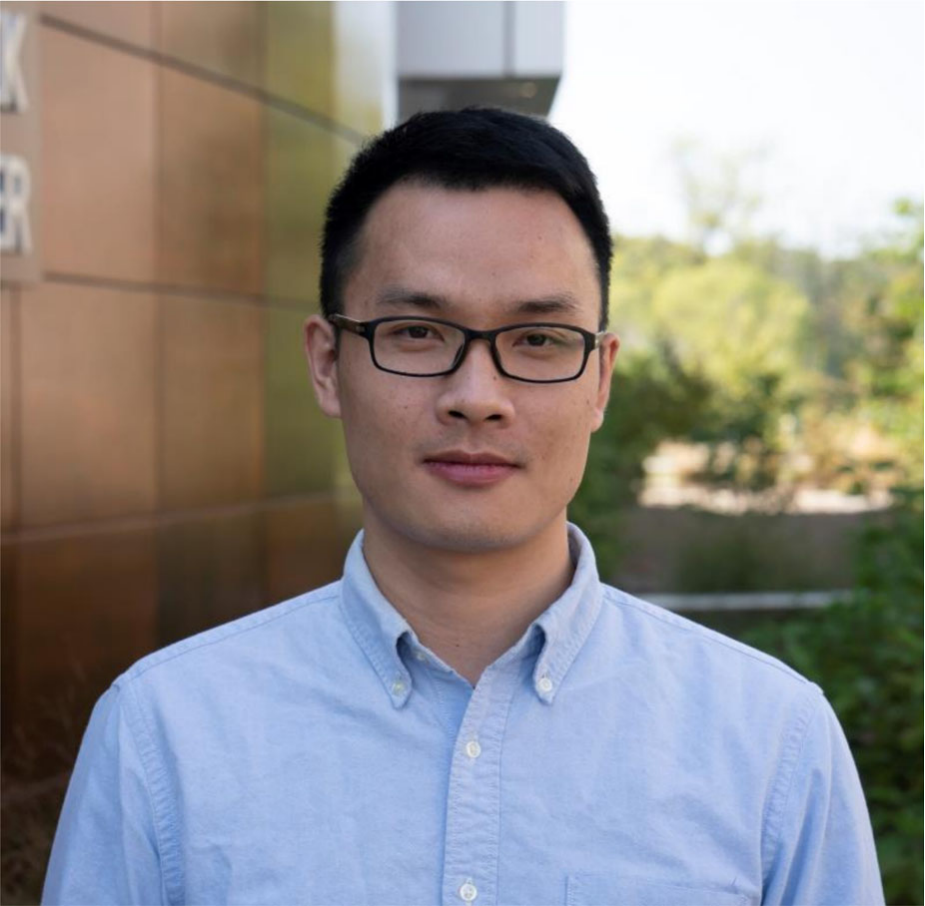

**Q1: Could you provide an overview of your scientific background and research experience?**

**What are your future research goals and aspirations?**

A1: I got my Bachelor of Science in Physics and Doctor of Philosophy in Optics, both from Zhejiang University. During my PhD, I spent half a year in the Chinese University of Hong Kong as an exchange student. Before joining Westlake, I was a research scientist at Purdue.

In my early career, I was interested in near-field quantum optical effects, such as the Casimir effect and the Super-Planckian effect. The Casimir effect, for example, describes how quantum fluctuations in vacuum lead to electromagnetic interactions that otherwise do not exist between charge-neutral objects according to classical physics. I focused on exploring those effects and their new features in novel optical meta-material systems. One of the interesting findings was the Casimir transport effect, where nanoparticles in quantum levitation could be propelled by lateral Casimir forces, forming a nanoscale maglev.

In those 5 years at Purdue, my research was more about classical and quantum optical sensing, especially for thermal light sources, ranging from information theory, novel sensing modalities based on meta-surfaces, to quantum-inspired algorithms for information extraction from thermal light. One of my representative works was Heat-Assisted Detection and Ranging (HADAR)^1^, which utilized thermal photons and information theory to see through pitch darkness like broad daylight. It was also during the research that I explored the field of artificial intelligence (AI) and gained valuable expertise. I saw how AI could solve complicated physics problems, and how physics laws could predict AI performance. My future research plans are deeply rooted in these experiences.

On joining Westlake, I’m all set and ready to launch a research group focusing on HADAR and AI physics. How could we leverage AI to help discover new physics laws? How could we understand and improve AI with the help of physics? What are the classical and quantum limits of AI set by physics laws? These thought-provoking questions will serve as a compass, guiding the group into the next frontier of physics.

**Figure:**
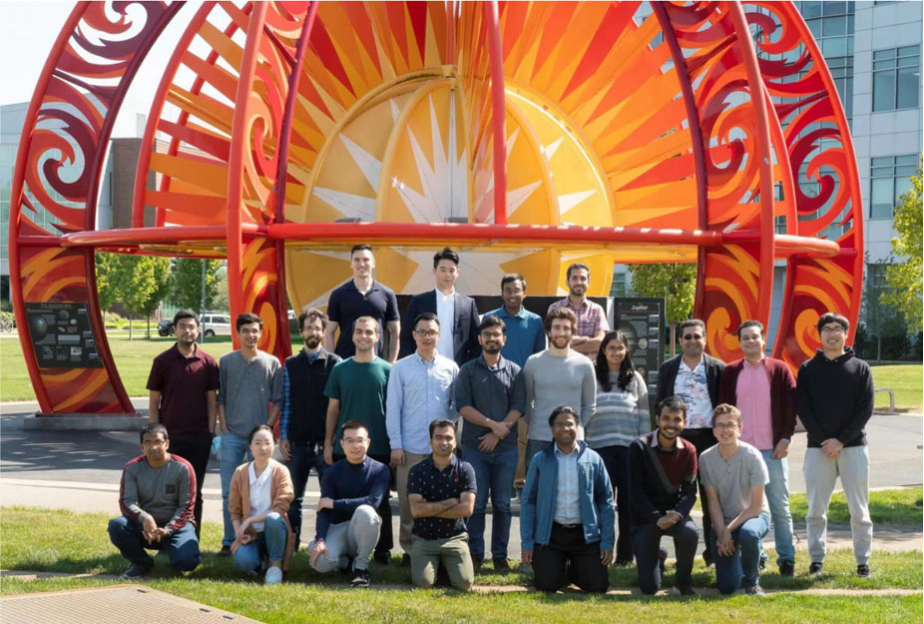
Dr. Fanglin Bao in Prof. Zubin Jacob’s group at Purdue

**Q2: What inspired you to delve into the world of science? What do you think are the most important attributes a great scientist should have?**

A2: During my time as a primary and middle school student, I did not merely extract great pleasure from playing outdoors, but I also started to have a sense of aspiration. I had a vague picture in my mind that there was an old gentleman and a blackboard, and he could explain everything in the world with only a piece of chalk. I hoped one day I could be that mentor. As I embarked on my college journey, I realized it was impractical for an individual to fully grasp every field, given the explosive growth of information. However, it is the tireless efforts of scientists that propel the boundaries of this expanding knowledge. I think it’s the strength of conviction that somehow has now led me to become a young scientist.

That being said, the course of being a scientist is never smooth. It is sometimes beset with hardships and obstacles and nothing short of a persistent spirit could help surmount them. It is sometimes filled with twists and turns, but the thirst for knowledge drives me forward. I am eager to ask the right questions and to discover more that could benefit the world.

Looking up to those great scientists in the past, it’s not difficult to notice that they all faced scientific difficulties with honesty. For instance, Max Planck confessed that he couldn’t fully understand the concept of “quantum” even after he proposed quantized energy of the electromagnetic radiation field. As Richard Feynman put it: “Never fool yourself, and remember that you are the easiest person to fool”, only when you are honest with science, could you discover the truth of the universe.

In a nutshell, aside from honesty, conviction, consistency, creativity, and curiosity all contribute to the success and impact of great scientists.

**Q3: Your scientific research paper has become the latest cover article of** ***Nature*, the world’s leading multidisciplinary science journal. How did you come up with the novel idea—Heat-Assisted Detection and Ranging (HADAR)? What specific scientific techniques or methodologies do you utilize in your work? How do you approach experimental design and data analysis in your research? How did you handle unexpected results during your research?**

A3: The work of HADAR covers almost the whole time I spent at Purdue. Interestingly, the term “Heat-Assisted Detection and Ranging” and the acronym “HADAR” had been invented even before I went to Purdue. In the first week I moved to West Lafayette, Zubin Jacob, my advisor, wrote down the word “HADAR” on one piece of draft paper in his office and explained to me a small project in his mind. Perhaps like most people, I was shocked a little bit by the term. Let’s be honest, “HADAR” is a nice name.

By then, none of us were experts in infrared remote sensing. Zubin’s original idea was to use the infrared radiation spectrum to classify materials with the help of machine learning. The advantage of such an original “HADAR” over traditional “LiDAR” (Light Detection and Ranging), “RADAR” (Radio Detection and Ranging), or “SONAR” (Sound Navigation and Ranging) was supposed to be its material information. The initial plan was for me to complete this start-up project within a 3-month timeframe, all while adapting to the new foreign environment.

Very soon, I found the idea to classify materials with infrared spectrum was reported in some literature many years ago. A couple of questions popped up in my mind: “Shall we give up this small project? Can we just add to it the functions of detection and ranging to close the case and move on to new projects? Wait. How can we range?” Till now, I’m still glad that I persevered from the start and went the extra mile in my research. In infrared thermal imaging, the key is the long-standing “ghosting effect”, a well-known optical phenomenon that makes thermal images blurry and textureless, not suitable for stereo vision. Thermal ranging was exactly the bottleneck if we kept HADAR a passive modality and relied on stereo vision. The project was getting interesting now, and we decided to continue working on it beyond the first 3 months.

After some analyses, I was able to understand the ghosting effect and proposed the TeX vision to overcome it. TeX vision disentangles the physics quantities of temperature (T), emissivity (e), and texture (X) from cluttered heat signals, and visualizes them in a way that mimics daylight optical imaging. Overall, TeX vision becomes a night vision technique that can see through pitch darkness like broad daylight, and with recovered textures, HADAR ranging becomes possible based on TeX vision. TeX vision is the core of the final version of HADAR. And it took us 4 years to eventually complete this “small” project.

During those years, our discussions in the building of the Birck Nanotechnology Center, together with the contributions from all co-authors, put HADAR into its current shape.

Explicitly, to extract information from heat signals, I built an inverse physics model reflecting the important roles that physics quantities of T/e/X played in the signals. To efficiently decompose heat signals into T/e/X components, I further developed the TeX-SGD (semi-global decomposition) algorithm, and later on, we developed the machine-learning algorithm TeX-Net. Those algorithms produced all the TeX vision results we got so far. Furthermore, to extensively test our theory and algorithms, we required a database. I worked with some experts in computer graphics to incorporate infrared physics into Monte Carlo path tracing. It took us more than half a year to eventually build the world’s 1st long-wave infrared stereo- and hyperspectral imaging database—the HADAR database. Having established a practical working principle for HADAR, we shifted towards uncovering its fundamental limits. Therefore, I derived the information-theoretic bounds for both HADAR detection and HADAR ranging and verified their validity in describing AI performance.

We built HADAR prototype-1 in our lab and collaborated with more professionals for HADAR prototype-2. The week we had the field test and data collection at Purdue was certainly an indelible experience, but it also took me more than a month to meticulously plan and design the field test experiment. To show the advantages of HADAR in texture recovery, we needed proper scenes with different types of thermal textures, the length scale of which had to be consistent with the sensor resolution. I did careful calculations and examined possible outdoor scenes in the greater Lafayette, to eventually narrow down our focus to the right spots.

Our HADAR theory required a material library as the input. The material library was very useful to illustrate our idea, but it wasn’t easy to collect in real-world experiments. How could we implement HADAR without the experimental data of the material library? We got stuck in this difficulty for months. Ultimately, I came up with an idea to estimate the material library from the hyperspectral imaging data itself, so that the input was not needed anymore.

**Figure:**
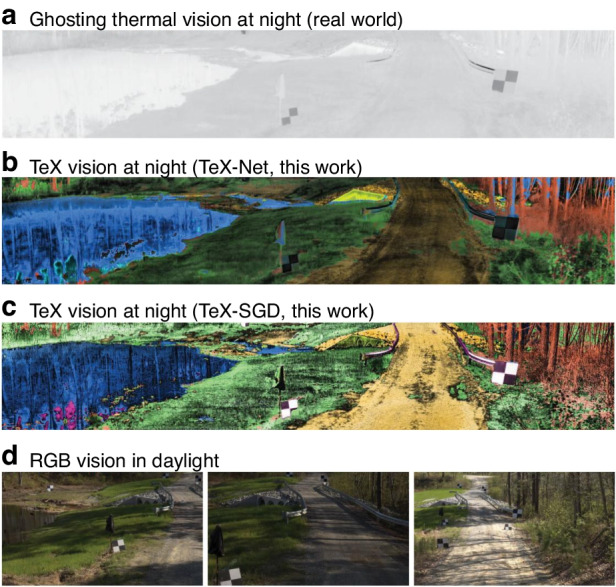
TeX vision at night *Nature*^1^

**Q4: To what aspects could your discovery be applied? Could you tell us more about its broader significance? What limitations does the study still have?**

A4: In the HADAR paper, we presented it as a paradigm shift in machine perception for artificial intelligence. Autonomous navigation is the first immediate application using HADAR’s night vision capability, where self-driving cars can navigate at night as if it were day. HADAR could also be used in smart healthcare for patient monitoring at night. Furthermore, HADAR could be used for wildlife monitoring, since most of the wild animals are active only at night. However, HADAR has far more advantages beyond night vision, as it can provide a comprehensive understanding of the scene. For example, HADAR can identify the material and accurately measure the temperature, beyond a human being’s ability. With access to extra physical information, HADAR is poised to facilitate revolutionary advancements in computer vision performance for AI.

Above all the explicit applications, the most striking implication of HADAR is that it inspires our rethinking of day and night. Through millions of years of evolution, we have been completely used to the dichotomy between day and night. But are nighttime and darkness inevitable? Could we turn night into day? With the assistance of AI, if we could have daylight vision 24 h a day, what life would be like? Do we still need lamps and streetlights? Our findings in HADAR spark this imagination. At least, there is one thing we are sure about: AI does not go through biological evolution, there is no reason for AI to suffer from the day-night dichotomy!

HADAR requires hyperspectral imaging in the long-wave infrared. This is extremely challenging in experimental implementations. Existing infrared hyperspectral cameras like Telops are as huge as a microwave oven, yet they take around 10 s to record one frame of fine-quality data. Such a slow response is insensitive to many daily events and common objects in motion. Furthermore, for the lack of big data, our machine learning algorithm, the TeX-Net, is largely restricted to synthetic data. Closing the domain gap between experimental scenes is crucial in order to harness the benefits of machine learning. We have to address and improve upon these limitations, and this is also one of my future research directions.

**Figure:**
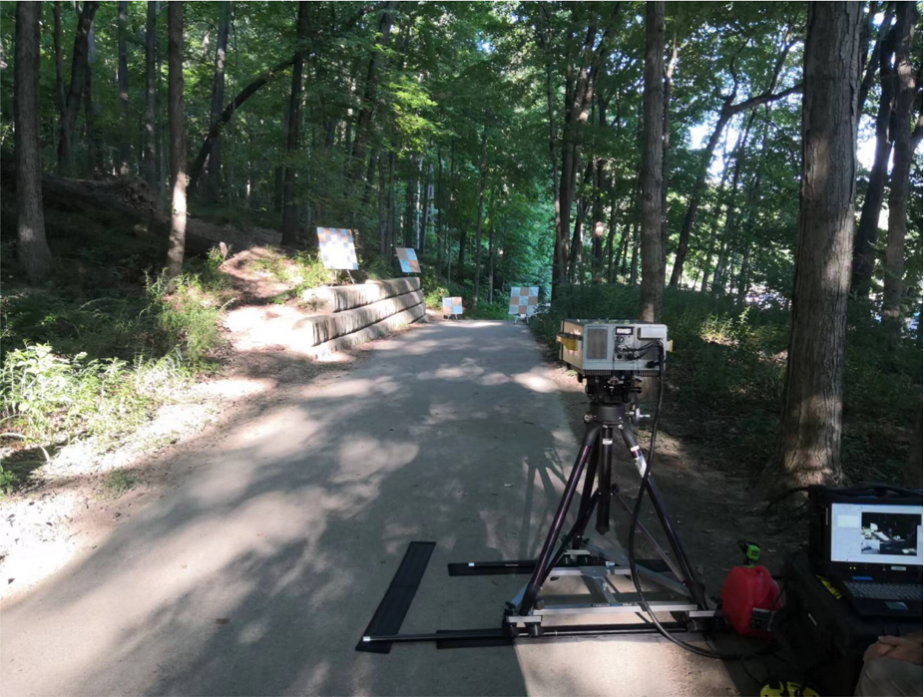
HADAR field test experiments in action

**Q5: What hobbies do you have after your research work? How would you strike the balance between work and life?**

A5: I like hiking and football, and with my family, I also enjoy playing badminton or watching movies. Balancing work and life had never been a problem until the birth of my daughter. In 2021, my daughter was born in the US during the COVID-19 pandemic. Unfortunately, she was allergic to eggs and milk. Taking care of her amidst the viruses and allergens was a big challenge for both my wife and me, especially when the daycare center was inaccessible. This is also the reason why we decided to move back to China, where we could easily access healthcare and receive more family support. With the little girl growing up healthily every day, I feel more energetic and eager to return to a normal research life.

**Figure:**
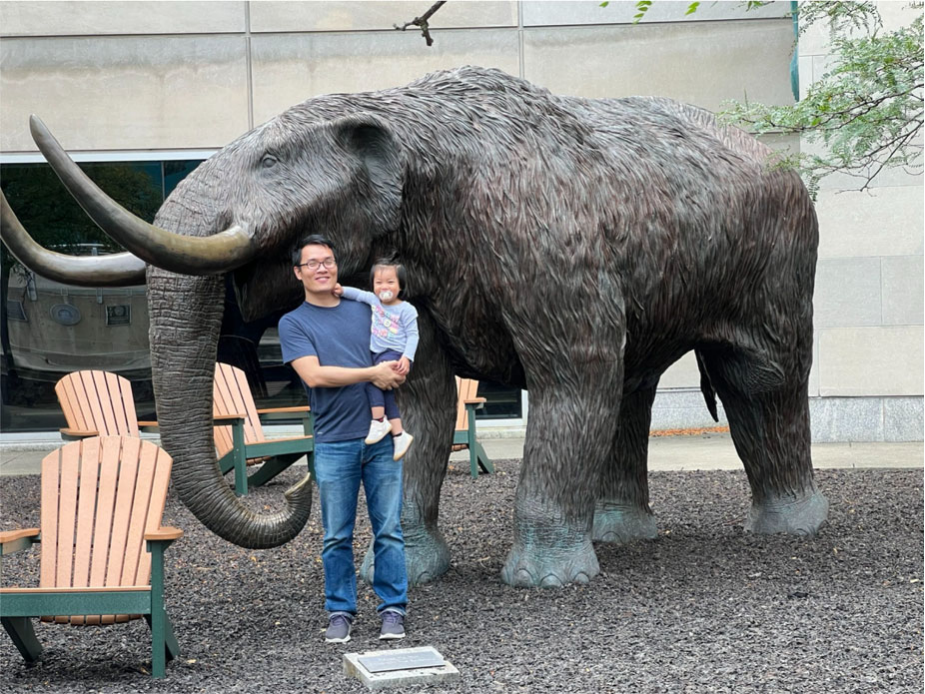
Father and daughter near Indianapolis Zoo

**Q6: In early 2024, you successfully joined the School of Science at Westlake, a top research institute in China. How has your experience been so far?**

A6: I had heard about Westlake University long before I went to Purdue as a post-doctoral scholar. During those years, I saw how Westlake grew from a concept to a real top university in China. Considering the esteemed reputation of Westlake, I could hardly imagine myself becoming a member of the institution, especially due to my multidisciplinary background, which slightly diverges from the traditional mainstream of physics. Typically, when applying for a faculty position, individuals with a multidisciplinary background may encounter greater difficulties or obstacles compared to those with a more specialized background. However, when I submitted the application, I soon found myself in a highly professional hiring process. I had my interview at Westlake University on Dec. 15, 2023, and joined the School of Science at Westlake on Jan. 15, 2024. It is truly impressive to witness how efficient Westlake is in making decisions and processing paperwork. And I’m also very glad that Westlake is friendly to new interdisciplinary research directions.

During the past month, I visited quite a few labs, talked to multiple PIs, and also walked around the gorgeous campus. I’m impressed by so many advanced facilities in operation, all in serene surroundings, where cutting-edge research is thriving. I enjoy the culture of Westlake as a research university centering around scientists.

Recently, I have also had the opportunity to interact with undergraduates through lectures. Unlike other universities in China, at Westlake, all lectures are delivered in English. Those young people show strong vitality and a deep understanding of the course. I am happy to realize that quite a few of them are at the level of students from the world’s top universities.

**Figure:**
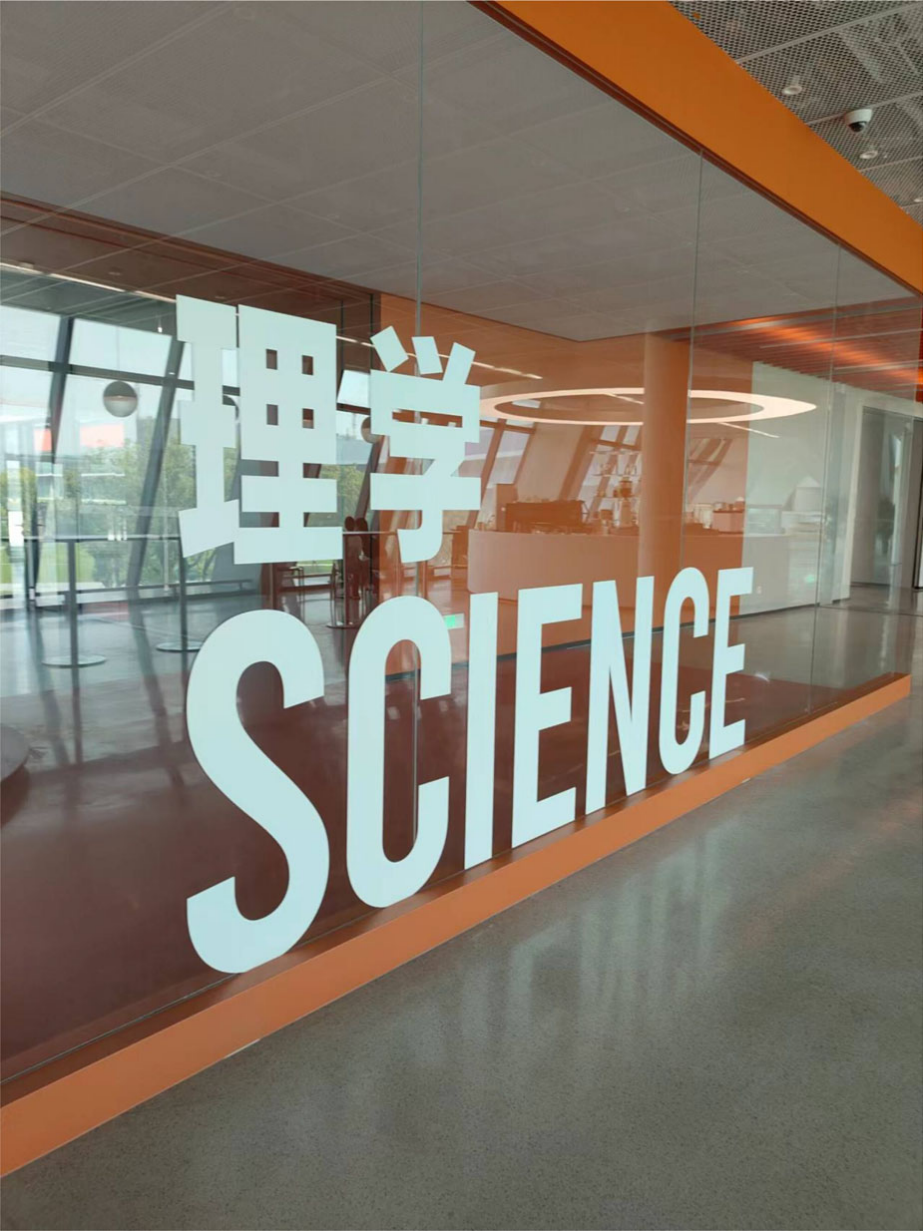
School of Science at Westlake

**Q7: In your eyes, what invites researchers or students at home and abroad to embark on an academic journey at Westlake?**

A7: Westlake is a very nice platform to conduct research, a platform led by many renowned scientists and constituted by competitive young faculties. Westlake’s strong support for young scientists sweeps away numerous trivial obstacles, enabling scientists to focus on research.

Westlake has an international background. Its call for our innovation and contribution to the world’s science and civilization is inspiring. I believe Westlake is a suitable platform for both Chinese and overseas scholars to develop their academic careers.

Such a hard-core environment is beneficial to students who want to learn scientific skills, especially those who want to pursue academic careers. The international environment and research experience in the world’s top labs serve as invaluable assets and credentials for students in their early careers.

**Q8: What advice would you give to today’s young students of science? What approaches or challenges would you take in nurturing the next generation of researchers?**

A8: I would say, please embrace critical and independent thinking; please be brave when you are doing something that no one else has ever done before and when no one can accompany you.

As my group is going for AI physics, I would like to use a simple example of machine learning to illustrate my philosophy of education. In machine learning, there are two main approaches: supervised learning and unsupervised learning. Supervised learning provides lots of question & answer pairs and gives explicit guidance on what actions to take, yielding high accuracy. However, it is considered the most elementary and falls short of our perception of true intelligence. On the other hand, unsupervised learning operates without the use of ground truth. Though it requires more advanced training strategies, its trained models can greatly impress. I would supervise in an “unsupervised” approach.

**Figure:**
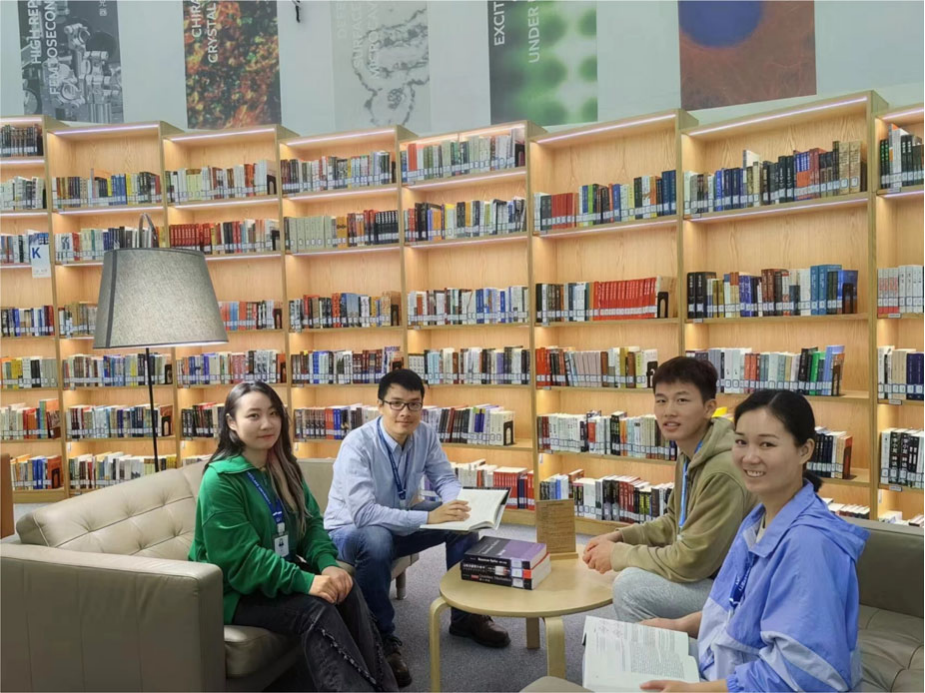
Prof. Fanglin Bao and his group at Westlake

**Q9: How do you stay updated with the latest developments and advancements in your field?**

**What topics do you believe would be featured as cover stories in prestigious journals like** ***Nature*?**

A9: To keep updated in my field, I read papers, attend workshops, and discuss with collaborators. I also like reading books extensively. Clever minds and their unique perspectives, even from different fields, can inspire me in my work and life. However, Once settled in a field, I would like to focus on my own ideas.

According to my humble experience, all papers accepted by *Nature* on various topics have the same chance of winning the cover story. Per *Nature*’s policy, the cover illustration is selected more for its aesthetic appeal than for its scientific content. In preparing the cover image, nevertheless, I did gain a few insights that I would like to share. The cover image and the cover story are anticipated to captivate a wide audience, including the non-scientific general public, in the most direct and inclusive manner possible. When examining previous cover images of top journals such as *Nature* or *Science*, readers cannot help but appreciate their exquisite blend of art and science. Moreover, these captivating visuals entice them to delve into the contents at a mere glance. The reason why an eye-catching cover image is difficult to select is that scientists usually live in the “momentum space”, an underlying world where jargon is daily language and lovely cats are freaky, while the general public lives in the real world. It is crucial to establish a connection between the abstract findings in the “momentum space” and the tangible implications they have in the real world.

In this sense, starting from the topics that are of great interest to the general public makes it a lot easier to dig into the “momentum space” and to travel back with novel scientific findings. In addition to the night vision technique we presented, I have read recent cover stories discussing diverse topics such as the formation of young stars, the extinction of the 3-meter-tall great ex-ape that once inhabited China, and the influence of air pollution by wildfires.

**Figure:**
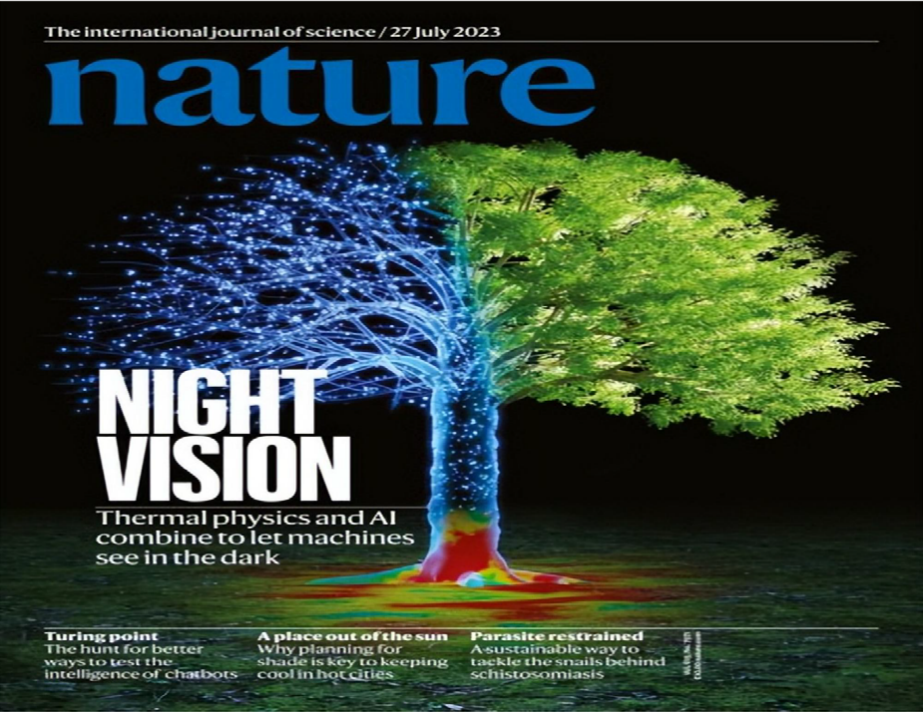
HADAR highlighted as *Nature* cover article *Nature*^1^

**Q10: How do you communicate your research findings to both scientific and non-scientific audiences?**

A10: In the past, I primarily disseminated my research findings through academic conferences, which is a crucial avenue for scientific communication. However, as the research moves forward, I intend to establish such platforms as blogs and personal web pages to reach a wider audience, including those beyond the scientific community.

First and foremost, these platforms provide an opportunity to communicate our research in a more audience-friendly manner. By presenting our findings in a language that is easily understood by the common people, we could bridge the gap between science and the general public, fostering greater awareness and understanding of our work. Secondly, blogs and personal web pages also allow for a more dynamic and interactive exchange of ideas. They provide a space where readers could engage in discussions, ask questions, and offer their insights, thereby facilitating a more inclusive and collaborative approach to the popularization of science. This two-way communication enables us to gain valuable perspectives from individuals with diverse backgrounds and expertise.

**Light special correspondent**

*Shirley Ji Wang，English Language Support Coordinator at School of Science, Westlake University.*
